# Predicting postpartum hemorrhage in women undergoing planned cesarean section: A multicenter retrospective cohort study in Japan

**DOI:** 10.1371/journal.pone.0306488

**Published:** 2024-07-09

**Authors:** Tomoko Yamaguchi, Hyo Kyozuka, Momoka Ito, Tsuyoshi Hiraiwa, Tsuyoshi Murata, Misa Sugeno, Fumihiro Ito, Daisuke Suzuki, Toma Fukuda, Shun Yasuda, Fujimori Keiya, Yasuhisa Nomura

**Affiliations:** 1 Department of Obstetrics and Gynecology, Ohta Nishinouchi Hospital, Koriyama city, Fukushima, Japan; 2 Department of Obstetrics and Gynecology, Iwase General Hospital, Sukagawa city, Fukushima, Japan; 3 Department of Obstetrics and Gynecology, Shirakawa Kosei General Hospital, Shirakawa, Fukushima, Japan; 4 Department of Obstetrics and Gynecology, Fukushima Medical University School of Medicine, Fukushima, Japan; Nazarbayev University School of Medicine, KAZAKHSTAN

## Abstract

Given Japan’s unique social background, it is critical to understand the current risk factors for postpartum hemorrhage (PPH) to effectively manage the condition, especially among specific groups. Therefore, this study aimed to identify the current risk factors for PPH during planned cesarean section (CS) in Japan. This multicenter retrospective cohort study was conducted in two tertiary maternal-fetal medicine units in Fukushima, Japan and included 1,069 women who underwent planned CS between January 1, 2013, and December 31, 2022. Risk factors for PPH (of > 1000 g and > 1500 g) were assessed using multivariate logistic regression analysis, considering variables such as maternal age, parity, assisted reproductive technology (ART) pregnancy, pre-pregnancy body mass index (BMI), uterine myoma, placenta previa, gestational age at delivery, birth weight categories, and hypertensive disorders of pregnancy (HDP). Multivariate linear regression analyses were conducted to predict estimated blood loss during planned CS. ART pregnancy, a pre-pregnancy BMI of 25.0–29.9 kg/m^2^, and uterine myoma increased PPH risk at various levels. Maternal smoking increased the risk of >1500 g PPH (adjusted odds ratio: 3.09, 95% confidence interval [CI]: 1.16–8.20). Multivariate linear analysis showed that advanced maternal age (B: 83 g; 95% CI: 27–139 g), ART pregnancy (B: 239 g; 95% CI: 121–357 g), pre-pregnancy BMI of 25.0–29.9 kg/m^2^ (B: 74 g; 95% CI: 22–167 g), uterine myoma (B: 151 g; 95% CI: 47–256 g), smoking (B: 107 g; 95% CI: 13–200 g), and birth weight > 3,500 g (B: 203 g; 95% CI: 67–338 g) were associated with blood loss during planned CS. Considering a patient’s clinical characteristic may help predict bleeding in planned CSs and help improve patient safety.

## Introduction

Planned cesarean section (CS) is a common obstetric intervention in which postpartum hemorrhage (PPH) is a well-known and life-threatening complication. Massive PPH is the most common cause of maternal death worldwide [1, 2], with life-threatening, massive PPH occurring in one out of every 300 cases [3]. PPH not only affects the immediate maternal prognosis but is also known to increase the risk of postpartum depression, and negatively impact breastfeeding through the development of anemia [4]. Therefore, reducing intraoperative bleeding is of significant importance.

Classically, PPH has been defined as blood loss > 500 g in vaginal delivery and > 1000 g in cesarean delivery [5, 6]. Recent studies have shown that the rate of cesarean deliveries has increased worldwide [7]. According to data published by the largest cohort study performed in Japan in 2014, 20.6% of all live births involved CSs [8]. The study also showed that the rate of CS increased with maternal age (15.2% among those aged 20–29 years and 34.7% among those aged > 40 years) [8]. In Japan, between 1990 and 2015, the proportion of pregnant women with advanced maternal age (AMA) increased from 7.7% to 29.0% [9]. These trends, which have also been observed in other developed countries, may reflect progress in the perinatal management of maternal complications such as uterine myoma and advances in assisted reproductive technology (ART) [9, 10]; therefore, CS rates will likely continue to increase. As the prevalence of CS increases, obstetric care providers will need to identify factors affecting PPH risk, as well as take recent changes in societal characteristics such as increasing maternal age and proportion of ART pregnancies into consideration, to provide optimal clinical management to patients. For those at high risk of PPH, careful diagnostic and intraoperative management strategies before CS can reduce the risk of morbidity.

Although many studies have analyzed risk factors for bleeding during CS [11–15], they failed to differentiate between scheduled and emergency CSs. Notably, emergency CSs, in contrast to elective ones, are frequently associated with obstetric complications such as placental abruption and labor arrest, which could potentially affect the factors contributing to PPH [16, 17]. Therefore, it is important to assess the PPH risks in cesarean sections by distinguishing between elective and emergency procedures, to estimate the risk of PPH more accurately. This study aimed to identify risk factors for PPH in planned CSs performed in Japan, with the goal of improving diagnostic and intraoperative management strategies to reduce morbidity risk associated with PPH.

## Methods

### Patients

This multicenter retrospective cohort study was conducted in two tertiary maternal-fetal medicine units in Fukushima Prefecture, Japan [16]. The study population consisted of pregnant women who delivered at either of these two units between January 1, 2013, and December 31, 2020. Vaginal deliveries, multiparous cases, and cases with insufficient data were excluded. The patients undergoing emergency CS were excluded. In our institution, planned CS were performed for cases with placenta previa, a previous history of cesarean section, or breech presentation. In this study, cases of placenta previa were excluded from the analysis, as it is already a well-known risk factor for PPH [17].

### Statement of ethics

This study was approved by the Institutional Review Board (IRB) of Ohta Nishinouchi Hospital (No. 37). The requirement for informed consent was waived by the IRB of Ohta Nishinouchi Hospital (No. 37) due to the retrospective nature of the study and the use of anonymized data. All procedures were performed in accordance with the ethical standards of our institutional and/or national research committee and the 1964 Helsinki Declaration and its later amendments or comparable ethical standards.

### Maternal and neonatal information

Maternal and neonatal data were extracted from the medical records of the included women who visited each medical unit. The data accessed for this research were collected between January and February 2023. The following maternal data were collected and used as covariate variables: AMA, prior births, method of conception, maternal body mass index (BMI), placenta previa, uterine myoma, and gestational age. AMA at delivery was defined as a maternal age ≥ 30 years, a value that was based on a previous Japanese epidemiological study [9]. Participants were categorized as primiparas or multiparas based on their history of delivery. The method of conception was dichotomized as the presence or absence of ART use, where ART pregnancy was defined as conception after *in vitro* fertilization and intracytoplasmic sperm injection or cryopreservation and frozen or blastocyst embryo transfers [18–20]. Maternal BMI was calculated according to World Health Organization standards (body weight [kg] / height^2^ [m^2^], kg/m^2^). Participants were categorized into the following six groups (G), according to pre-pregnancy BMI: G1, < 18.5 kg/m^2^; G2, 18.5 to < 20.0 kg/m^2^; G3, 20.0 to < 23.0 kg/m^2^; G4, 23.0 to < 25.0 kg/m^2^; G5 (25.0 to < 30.0 kg/m^2^), and G6 (≥ 30.0 kg/m^2^) [16, 21–23]. In the study setting, cervical length was routinely measured between 28 and 30 weeks of gestation, with placenta previa defined when the placenta completely covered the internal os of the cervix. In contrast, the placental position was defined as normal when the placental-cervical distance was > 2 cm [24]. Uterine myoma was confirmed if any myoma was present at the time of CS. Gestational age was determined during the early stages of pregnancy based on the mother’s last menstrual period and/or ultrasound findings.

Birth weight was measured by a midwife immediately after delivery. Patients were categorized into four groups based on infant birth weight, as follows: < 2500 g; 2500–2999 g; 3000–3499 g, and > 3500 g. In this study, hypertensive disorders of pregnancy (HDP) were defined as chronic maternal or new-onset hypertension after 20 weeks of gestation [25]. Estimated blood loss was also defined based on the contents of the suction jar in the operating room and the weight of surgical pads. PPH was defined as an estimated blood loss of > 1000 g [5] and an additional subcategory was defined for cases in which the blood loss was > 1500 g. For the present analysis, we assumed that one gram of blood was equivalent to one milliliter. In this study, we included factors that had previously been associated with obstetric bleeding as well as variables identified in our data set as confounding factors [9, 19, 26].

### Statistical analysis

No statistical sample size calculation was conducted because of its retrospective nature. Data of women who underwent CS were reviewed. Patients were categorized into two groups, as follows: those with PPH > 1000 g, and those without PPH. Maternal medical backgrounds and obstetric outcomes of groups were compared. The chi-square test was used to compare categorical variables and the t-test was used to compare continuous variables after confirming that each continuous variable was normally distributed. To evaluate the risk factors for PPH (> 1000 g and > 1500 g), multivariate logistic regression analysis was conducted, which included the following variables: maternal age (AMA or not), parity (primipara or multipara), ART pregnancy, pre-pregnancy BMI (G1 to G6), uterine myoma, gestational age at delivery (weeks), birth weight (four categories), and HDP. Both univariate and multivariate linear analyses were conducted using the same confounders, with the amount of estimated blood loss during CS as a continuous variable outcome. In the linear regression analysis, the results were presented as B: Partial regression coefficients and 95% confidence intervals (CI). Dummy variables were used for categorical variables that consisted of more than three categories. In the multiple regression analysis, the variance inflation factor (VIF) was calculated for each independent variable, and any variable with a VIF above 5 was excluded from the subsequent analysis. SPSS version 26 (IBM Corp., Armonk, NY, USA) was used to perform all statistical analyses. The level of statistical significance was set at p < 0.05.

## Results

**Fig 1:**
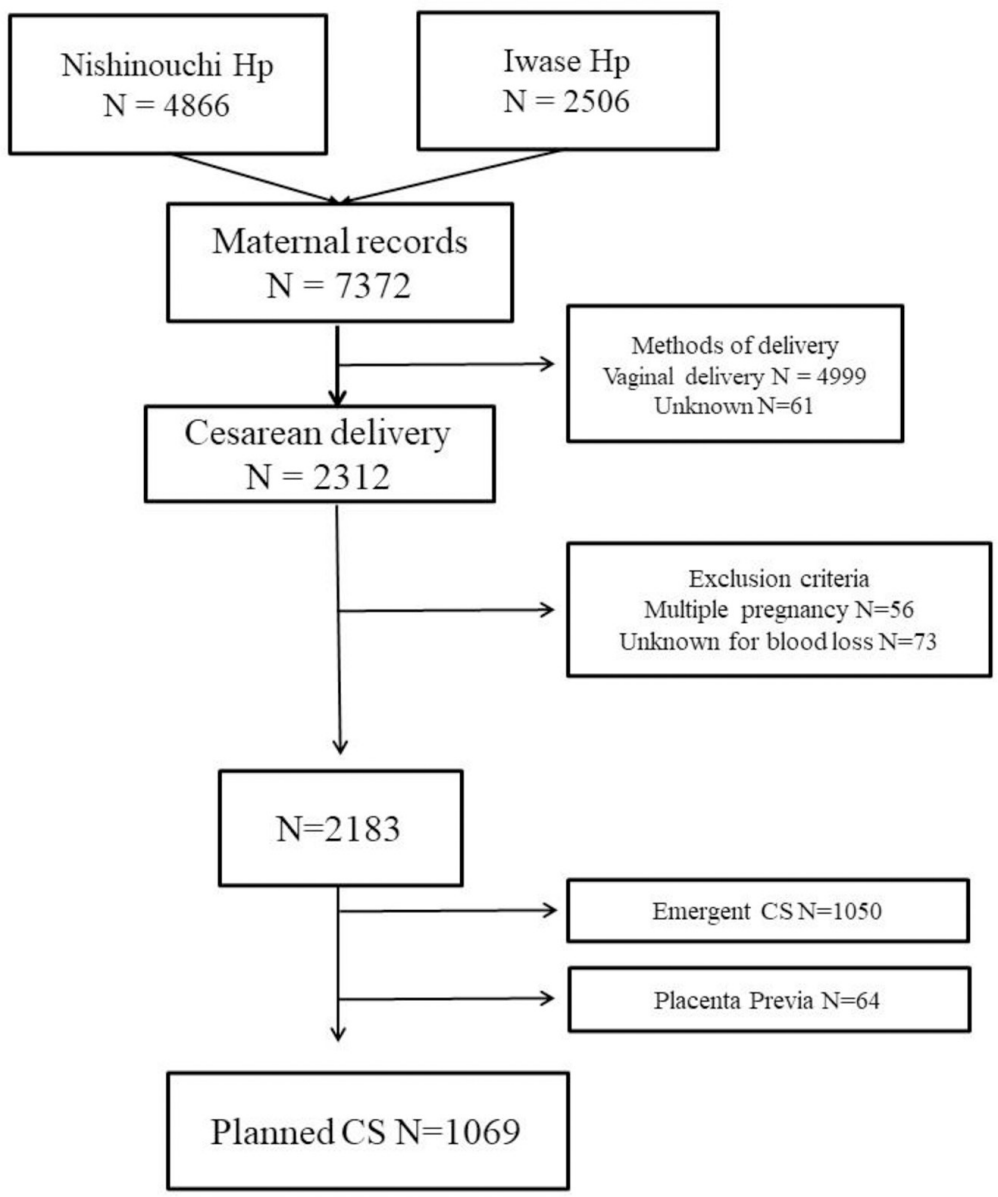
Study flow.

A flow chart depicting the process of participant selection is shown in Fig 1. Throughout the study period, 4866 and 2505 patients were treated at Nishinouchi and Iwase Hospitals, respectively. Sixty-four cases of placenta previa were excluded. After applying the inclusion criteria, 1,069 cases of planned CSs were included in the study. No patient required cesarean hysterectomy.

Maternal histories and obstetric outcomes of patients with and without PPH are reported in Table 1. Among all planned CSs, 18.1% (193/1069) of the cases involved PPH. Regarding maternal background, when cases with and without PPH were compared, a significant difference was observed in terms of AMA proportion (81.7 vs. 71.3, p < 0.001), and the presence of uterine myoma (14.0% vs. 4.7%, p < 0.001). Regarding obstetric outcomes, the mean infant birth weight was significantly higher in patients with PPH than in those without (2,973 g vs. 2744 g, p < 0.001).

**Table 1:** Basic characteristics of participants with and without PPH.

Variable	PPH (+) N = 193	PPH (-) N = 876	p-value
Maternal age, mean years (SD)	33.7 (4.6)	32.4 (5.0)	< 0.001^a^
Advanced maternal age, %	81.7	71.3	0.003^b^
Primiparous, %	29.0	21.2	0.019^b^
ART pregnancy, %	6.2	3.5	0.086^b^
Maternal smoking, %	7.8	7.7	0.942^b^
BMI, kg/m^2^, %			
< 18.5	9.3	10.8	0.006^b^
18.5–19.9	15.9	17.5	
20.0–22.9	28.0	36.4	
23.0–24.9	11.0	12.5	
25.0–29.9	24.2	13.1	
≥ 30.0	11.5	9.6	
Uterine myoma, % (n)	14.0	4.7	< 0.001^b^
Obstetrics outcome			
Gestational age, mean weeks (SD)	37.6 (0.8)	37.3 (1.7)	0.018^a^
Birth weight, mean g (SD)	2973 (393)	2744 (471)	< 0.001^a^
Birth weight, g, %			
< 2500	8.3	22.3	< 0.001^b^
2500–2999	47.2	50.5	
3000–3499	36.8	24.2	
≥ 3500	7.8	3.1	
HDP, % (n)	4.1	6.5	0.214^b^

SD, standard deviation; PPH, postpartum hemorrhage; ART, assisted reproductive technology; BMI, body mass index; HDP, hypertensive disorders of pregnancy

^a^ p-value from t-test

^b^ p-value from the chi-square test

p < 0.05 is statistically significant

Table 2 shows risk factors for PPH of > 1000 g and of > 1500 g. Maternal age more than 30, pre-pregnancy BMI of 25.0–29.9 kg/m^2^, and uterine myoma increased the risk of PPH > 1000 g (adjusted odds ratio [aOR]: 1.66, 95% confidence interval [CI]: 1.08–2.57; aOR: 2.42, 95% CI: 1.49–3.96; and aOR: 2.71, 95% CI: 1.51–4.85; respectively). Maternal smoking, pre-pregnancy BMI of 25.0–29.9 kg/m^2^, and uterine myoma increased the risk of PPH > 1500 g (aOR: 3.09, 95% CI: 1.16–8.20; aOR: 2.80, 95% CI: 1.08–7.23; and aOR: 4.62, 95% CI: 1.89–11.3; respectively).

**Table 2:** Risk factors for PPH > 1000 g, and 1500 g.

Variable	PPH > 1000g				PPH > 1500g			PPH > 2000g		
	aOR		95% CI	P value	aOR	95% CI	P value	aOR	95% CI	*P* value
Advanced maternal age	1.74		1.14 to 2.65	0.01	1.89	0.83 to 4.30	0.13	1.89	0.35 to 10.1	0.46
Multipara	0.73		0.49 to 1.09	0.12	0.93	0.45 to 1.92	0.852	1.14	0.27 to 4.85	0.86
ART pregnancy	2.34		1.17 to 4.67	0.016	2.83	1.03 to 7.82	0.044	10.4	1.78 to 60.7	0.009
Maternal smoking	1.44		0.78 to 2.67	0.244	**2.63**	**0.99 to 7.03**	**0.053**	11.6	2.85 to 46.9	0.001
BMI, kg/m2										
<18.5-	0.92		0.53 to 1.77	0.971	0.32	0.07 to 1.49	0.146	2.95	0.22 to 39.3	0.414
18.5–19.9	1.16		0.71 to 1.90	0.923	1.89	0.83 to 4.30	0.128	7.69	1.06 to 55.9	0.044
20.0–22.9	Ref		-	-	Ref	-	-	Ref	-	-
23.0–24.9	1.12		0.64 to 1.95	0.687	1.33	0.50 to 3.54	0.573	14.8	2.05 to 107.2	0.008
25.0–29.9	**2.27**		**1.41 to 3.66**	**0.001**	**2.47**	**1.08 to 5.68**	**0.033**	13.1	1.65 to 1003.9	0.015
≥30	1.06		0.57 to 1.97	0.857	1.1	0.35 to 3.45	0.87	8.13	0.83 to 80.0	0.073
**Uterine myoma**	**2.71**		**1.51 to 4.85**	**0.001**	5.33	2.20 to 12.9	<0.001	NA	-	0.997
Placenta previa	8.79		4.71 to 16.4	<0.001	18.8	8.19 to 43.3	<0.001	33.8	8.58 to 133.5	<0.001
Gestational age, week	0.95		0.82 to 1.10	0.506	0.79	0.62 to 1.02	0.069	0.57	0.32 to 1.03	0.064
Birth weight										
<2500	0.24		0.13 to 0.45	<0.001	0.12	0.03 to 0.43	0.001	0.02	0.00 to 3.26	0.13
2500–2999	0.56		0.38 to 0.81	0.002	0.39	0.20 to 0.76	0.006	1.49	0.35 to 6.31	0.592
3000–3499	Ref		-	-	Ref	-	-	Ref	-	-
≥3500	1.54		0.72 to 3.30	0.267	2.23	0.74 to 6.74	0.156	7.21	0.88 to 59.0	0.066
HDP		0.65	0.29 to 1.46	0.292	0.16	0.02 to 1.72	0.132	0.45	0.02 to 10.7	0.622

PPH: postpartum hemorrhage; aOR: adjusted odds ratio; CI: confidential interval; ART: assisted reproductive technology; BMI: body mass index; HDP: hypertensive disorders of pregnancy; Ref: reference; NA: not available

Factors related to estimated blood loss, treated as a continuous variable outcome in planned CSs, were calculated via univariate and multivariate linear analyses and are presented in Table 3. Multivariate linear analysis revealed that AMA (B: 83 g; 95% CI: 27–139 g), ART pregnancy (B: 239 g; 95% CI: 121–357 g), maternal smoking (B: 107 g; 95% CI: 13–200 g), pre-pregnancy BMI of 25.0 to –29.9 kg/m^2^ (B: 74 g; 95% CI: 22–167 g), uterine myoma (B: 151 g; 95% CI: 47–256 g), and birth weight > 3500 g (B: 203 g; 95% CI: 67–3338 g) were associated with increases in the estimated blood loss during planned CS.

**Table 3:** Factors related to estimate blood loss during planned cesarean section determined via univariate and multivariate linear regression analyses.

Variable	Univariate linear regression analysis			Multiple linear regression analysis^1^		
	B (g)	95% CI	p-value	B (g)	95% CI	p-value
Advanced maternal age	94	43 to 145	<0.001	83	27 to 139	0.004
Multipara	−57	−111 to −4	0.036	−49	−109 to 10	0.104
ART pregnancy	78	−37 to 194	0.183	239	121 to 357	<0.001
Maternal smoking	65	−19 to 150	0.127	107	13 to 200	0.026
BMI, kg/m^2^						
< 18.5-	−32	−105 to 46	0.421	−0.1	−84 to 84	1.000
18.5–19.9	−16	−79 to 47	0.615	26	−45 to 97	0.473
20.0–22.9	Ref	−	−	Ref	−	-
23.0–24.9	19	−54 to 92	0.614	58	−24 to 139	0.164
25.0–29.9	79	13 to 146	0.019	74	22 to 167	0.011
≥ 30	49	−31 to 128	0.231	11	−81 to 102	0.815
Uterine myoma	266	174 to 357	<0.001	151	47 to 256	0.005
Gestational age, week	26	12 to 41	<0.001	−21	−40 to −3	0.026
Birth weight						
< 2500	−178	−238 to −122	< 0.001	−244	−324 to −164	<0.001
2500–2999	−23	−69 to 22	0.312	−95	−156 to −36	0.002
3000–3499	Ref	−	−	Ref	−	-
≥ 3500	251	135 to 367	<0.001	203	67 to 338	0.004
HDP	−135	−229 to −40	0.005	−145	−254 to −37	0.009

PPH, postpartum hemorrhage; B, partial regression coefficient; aOR, adjusted odds ratio; CI, confidence interval; ART, assisted reproductive technology; BMI, body mass index; HDP, hypertensive disorder of pregnancy.

^1^The R-squared value in the linear regression analysis was 0.12

## Discussion

Our study identified several established risk factors for PPH among women undergoing planned CS, including AMA, uterine myoma, maternal smoking, and high maternal BMI. Importantly, although ART was not a risk factor for PPH, defined either as blood loss >1000 g or >1500 g, in multiple logistic regression analysis, multiple linear regression analysis indicated that an ART pregnancy was the factor that affected blood loss the most during planned CS, followed by birth weight > 3,500 g, uterine myoma, maternal smoking, maternal age > 30 years, and maternal pre-pregnancy BMI of 25.0 to < 30.0 kg/m^2^. These findings suggest that obstetric care providers should consider the potential risk associated with ART pregnancy when assessing risk of PPH in women undergoing planned CS.

Our findings suggest that smoking during pregnancy is a significant risk factor for PPH development. The underlying mechanism linking smoking during pregnancy to abnormal bleeding remains unclear. One possibility is that systemic inflammation induced by air pollutants [27, 28] may impact the uterine endometrium, leading to poor decidualization [29]. Animal studies have shown that exposure to fine particulate matter (PM_2.5_) during pregnancy can lead to placental inflammation [30]. Thus, it is reasonable to assume that pollutant-induced inflammation during pregnancy could result in inflammation of the endometrium [31], leading to placental adhesion to the uterus. Consequently, maternal smoking may increase the risk of blood loss during CS due to abnormal placentation of the uterus [19].

The relationship between ART pregnancy and PPH can be attributed to abnormal placentation, as proposed by Esh et al. [32]. The pathogenesis of PPH due to ART may rely on mechanical factors such as primary deficiency in the decidua caused by local trauma to the uterine wall or biological factors such as an abnormal maternal response to trophoblast invasion. Abnormal placentation in ART pregnancies may result in the impaired development of spiral arteries, leading to placental adhesion to the uterus and subsequent PPH [19]. While few studies have examined the associations between ART treatment and maternal PPH risk, those that have all indicate that women who underwent ART are at higher risk of PPH [33–36]. However, our study is the first to focus solely on planned cesarean sections. Furthermore, ART is strongly associated with placenta previa, a well-known PPH risk factor [35]. By excluding cases of placenta previa, the present study demonstrates that there is also a direct relationship between ART pregnancies and PPH during planned CS, which is independent of the presence of placenta previa.

In recent years, developed countries have seen an increase in maternal age, which is associated with a heightened risk of obstetric complications [9]. This increase in maternal age suggests a rising demand for ART pregnancies [36]. Our results indicate the need to pay attention to the enhanced risks and provide specialized management approaches in cases of ART pregnancies, particularly in light of their escalating prevalence. This investigation offers essential insight for healthcare provider, providing them with the knowledge to foresee and mitigate the distinct risks inherent to ART more effectively. Consequently, the results presented here have the potential to significantly enhance maternal health outcomes in developed countries.

The prevention of PPH by minimizing its risk factors is a major clinical challenge. There has been increasing interest in the influence of preconception care on obstetric outcomes, including lifestyle modifications such as improving nutrition and smoking cessation [25, 37, 38]. Modifiable factors such as high maternal BMI and smoking status are significant risk factors for PPH. Therefore, preconception care focusing on improving daily maternal nutrition and smoking cessation are potential preventive strategies that may allow for the reduction of morbidity risk associated with PPH. It is important to emphasize the potential benefits of preconception care based on these factors due to their modifiable nature and potential for improving maternal and fetal outcomes. Promoting healthy preconception habits and emphasizing the importance of preconception care will likely improve maternal and fetal outcomes.

In the present study, unmodifiable risk factors for PPH including ART pregnancy, uterine myoma, and maternal age were found to contribute to massive PPH during planned CS. Although preventive strategies taken during preconception care will likely fail to minimize risk due to these factors, use of minimally invasive intraoperative management strategies such as resuscitative endovascular balloon occlusion of the aorta (REBOA) may provide a solution. REBOA has been shown to reduce overall blood loss and transfusions, improving maternal outcomes in patients with expected placenta accreta spectrum disorders [39–43]. Our institution recently introduced REBOA for high-risk PPH cases [44]. We consider that the use of REBOA may help prevent PPH when bleeding risk factors identified in our analysis coincide with placenta previa, a known bleeding factor. We are currently conducting a multi-institutional clinical study to determine whether the use of REBOA can reduce the risk of PPH in women with placenta previa who present with risk factors identified in this analysis.

A primary strength of the current study is that patient data were derived from two tertiary care fetal medicine units in which all patients were managed by obstetricians with equivalent training for obstetric emergencies [16]. Nonetheless, this study has potential limitations. First, the retrospective nature of the study may have allowed for the introduction of bias, and errors in data collection and analysis. Second, planned CS were performed for cases with a history of previous CSs and breech presentation. However, we did not stratify the cases by their specific indications for CS. Further analysis is required on how preoperative preparations vary according to different indications for planned CS.

In conclusion, this study aimed to predict risk of bleeding during planned CS by considering factors related to a patient’s social background such as maternal age and ART pregnancy. Our findings suggest that evidence-based practices that consider the social backgrounds of patients may improve patient safety during planned CS.

## Supporting information

S1 Data (TXT)

## List of abbreviations

AMA: Advanced maternal age
ART: Assisted reproductive technology
BMI: Body mass index
CI: Confidence interval
CS: Cesarean section
HDP: Hypertensive disorders of pregnancy
IRB: Institutional Review Board
JECS: Japan Environment and Children’s Study
PPH: Postpartum hemorrhage
REBOA: Resuscitative endovascular balloon occlusion of the aorta
